# A comparative study of severe thunderstorm among statistical and ANN methodologies

**DOI:** 10.1038/s41598-023-38736-z

**Published:** 2023-07-25

**Authors:** Sonia Bhattacharya, Himadri Chakraborty Bhattacharyya

**Affiliations:** 1 State Aided College Teacher, Department of Computer Science, Panihati Mahavidyalaya Barasat Road, Sodepur, Kolkata India; 2 Principal, Bangabasi College, University of Calcutta, UGC Sponsored visiting Faculty, Institutue of Radio Physics and Electronics, University of Calcutta, Kolkata, India

**Keywords:** Natural hazards, Engineering, Mathematics and computing

## Abstract

Severe Thunderstorms are the extreme weather convective features. It causes local calamities in various ways. Proper prediction with lead time is an important factor to prevent such calamities from saving people. Here, both probabilistic and machine learning techniques are applied to weather data to obtain proper predictions. Traditional methodologies are already available for such prediction purposes. However, Naïve Bayes and RBFN (Radial Basis Function Network) methodology have been introduced here with some specific weather parameters that has not done before remarkably. A comparative study was performed on weather data including Naïve Bayes, Multilayer Perceptron (MLP), K-nearest neighbor (KNN) and Radial Basis Function Network (RBFN). All these data have been procured from Kolkata located in north-east India. The result obtained by applying the Radial Basis Function Network is better among the three methods, yielding a correct prediction of 95% for severe “squall-storms” and 94% for “no storm”. The predictions have a sufficient lead time of 10- 12 h.

## Introduction

Thunderstorms are mesoscale convective processes that cause some extreme weather events. That may include suffering, heavy rainfall, hail and gusty winds^1^. Wind speed of at least 45 km/h having minimum duration of 1 min is called ‘squall’. Generally, a thunder-squall can persist for a maximum of one minute with a spatial extent of 100 kms ^2^. Since the thunderstorm is an extreme convective weather event, proper prediction is needed to alert the people who reside within the devastating region (100 km)^3^. Thunderstorms occur every year in pre monsoon season over North east India. The days that have a record of ‘squall’ wind has considered here as ‘thunderstorm days’. Similarly, the days that have no such records of ‘squall’ wind has considered here as ‘no thunderstorm’ days^4,5^. The main aim of this study is to forecast severe thunderstorm with an enough lead time by comparing among various ANN (Artificial Neural Network) methodologies i.e. Multilayer Perceptron (MLP), K-Nearest Neighbor (KNN) Method, Radial Basis Function Network (RBFN) and statistical methodology i.e. e. Naive Bayes Method. The life cycle of a thunderstorm has three stages: cumulus (updraft persisting throughout the cell), mature (presence of both the updraft and the downdraft), and dissipating (manifested only by the downdraft all through the cell)^6^. It is the towering cumulus or the cumulonimbus clouds of the convective origin and high vertical extent that are capable of producing lightning and thunder. The study revealed that every cumulus tower is sheared at a much lower rate than if it drifted with the wind^7^. Outlying cumuli are frequently torn as under when subjected to strong vertical shear^8^. Thunderstorms can be categorized as single cells, multicells, squall lines and supercells ^9^.Byers and Batton (1949) performed a study with the help of radar data. The simulation of mesoscale model is helpful to justify the physics and dynamics of the severe thunderstorms^3,10^. Mathematical physics establishes that primary value problem is mainly to forecast the state of the atmosphere, whereas future weather is predicted by integrating the governing partial differential equations, starting from the observed current weather^11^. Most weather prediction systems use a combination of empirical and dynamical techniques^12^. Thunderstorms forecasting is a complicated task in weather prediction. The reason behind this is the small spatial and temporal extension of thunderstorms and the inherent nonlinearity of their dynamics and physics^13^.The success and failure of predictions is exactly known and pathways to obtain better predictive skill can be efficiently tested^14,15^. Parameterizations play an important part in forecasting skill since they determine the main features of the simulated weather, such as clouds and precipitation^11^. Forecast consistency is determined by contrasting forecast circulations with the observed constancy of occurrence^16^. Convective Available Potential Energy (CAPE) value indicates the presence of updrafts, more the value more the possibility of severe thunderstorm. Convective Inhibition (CINE) is the energy that needs to be overcome in order for convection to occur^17^. The roles of CINE and CAPE have been studied for the forecasting purpose by many researchers^18^. Some weather parameters which have been considered here in this work are very much related to generate the CAPE by overcoming CINE. Researches also show that vertical velocity, relative humidity and wind shear, plays very vital role to form the severe thunderstorm^4^. Therefore other weather parameters have been considered here in this study to analyse their relations if any with the formation of severe thunderstorm. Methodologies have been developed to offer vital information on the probability of severe weather^19^. The Numerical Weather Prediction (NWP) model is a very useful tool for diagnosing the structure of thunderstorms. The applications of different NWP models on different weather parameters (such as vertical velocity, relative humidity and wind shear) yields promising result. The application of the NN model in the field of meteorology has been increasingly applied in meteorological research^20–24^. The application of a neural network that learns rather than analysing such compound relationships has revealed an immense deal of assurance in accomplishing the objective of weather forecasting with elevated accuracy^25–27^. The weather prediction reports require some intelligent calculations that can deal with the nonlinear dataset. This creates some rules and patterns to learn from the experimental data to forecast the weather in the future^28^. ANNs (Artificial Neural Networks) have the benefit of their skill to learn and become accustomed^27^. Gyanesh Shrivastava et al., revealed that BPN (Back-Propagation Neural Network) and RBFN (Radial Basis Function Network) are competent model for predicting monsoon rainfall. The forecast of monsoon rainfall based on artificial neural network is a well-researched problem^29^. These models are also effective for small range weather forecast. BPN and RBFN give suitable solutions for the prediction of long-range weather forecasting^30^. Chaudhuri et al., has showed in their studies the use of multilayer perceptron logic and fuzzy logic to analyse the role of different weather parameters for thunderstorm prediction purpose^24,31,32^.

The Multilayer Perceptron^33^ and KNN (K-Nearest Neighbor) all have been applied on different weather parameters (such as moisture data) for severe thunderstorm prediction purpose previously. There are many studies where statistical and different machine learning techniques have been applied on different weather parameters to predict severe thunderstorm. But here in this study two different methodologies have been introduced on others weather parameters for prediction purpose which are innovative. The results obtained from these methodologies have been compared here with the conventional methodologies (such as Multilayer Perceptron, KNN) also. Here the RBFN and Naive Bayes classifier are introduced for the severe thunderstorm prediction purpose. RBFN has not previously been used for this purpose. The Naive Bayes classifier has been chosen for lightning storm detection purposes using lightning data^34^. Li et al. (2019) applied Naive Bayes for sandstorm prediction purposes. Wu et al. (2015) used RBFN to predict rainfall forecasts, which gave 88.49% correct prediction. Surface temperature prediction has been done using RBFN with a good accuracy of Litta et al. (2015). However there is no benchmark study that predicts severe thunderstorms using RBFN and Naive Bayes using the mentioned weather data with a high accuracy level. The main aspect of this study is as follows:

- A less used methodology has been applied here which gives a high accuracy rate.
- A comparative study was performed among conventional methodologies (Multilayer Perceptron, KNN, Naive Bayes) and RBFN
- A comparative study also reveals that RBFN gives much more promising results than the others.
- This study has a lead time of 10–12 h which is very much important so that the government can take proper precautions to save life and property.

In this study six different weather parameters were considered for severe thunder storm prediction. These six weather parameters are cloud coverage, sunshine hours, pressure at the freezing level and three different dry adiabatic lapse rates at different geopotential heights of the atmosphere. Here different methodologies (both statistical and ANNs) have been applied to these weather parameters for prediction purposes^4,35^. The Naive Bayes classifier has been applied here as a statistical methodology to these weather parameters. This yields more than 85% correct prediction for ‘squall days’ and 86.34% correct prediction for ‘no squall days’. The application of the K Nearest Neighbour (KNN) method on the mentioned data set gives more than 88% correct prediction for ‘squall days’ and more than 87% correct prediction for ‘no squall days’. Then Multilayer Perceptron (MLP) has been applied on the six mentioned weather parameters which produce 91.8% correct prediction for ‘squall days’ and 89.27% correct prediction for ‘no squall days’. The most promising results emerge by the application of Radial Basis Function network (RBFN). This gives more than 95% correct prediction for ‘squall days’ and more than 94% correct prediction for ‘no squall days’ on the mentioned weather parameters. In case of a proper weather forecast correctness of a methodology is not the only one factor. A weather prediction without any lead time has no importance. Therefore in case of a proper weather forecast enough lead time is very much necessary. Here in this study a lot of importance has given to these matters. Lead time is the duration of time which is predicted before the onset of the occurrence of the event. The development of the thunderstorm generally begins from the early morning and occurs on the evening time. It can develop within the span of 10–12 h and then it occurs. The lead time is not only important to alert the people but also the Government for taking precautionary measures. All these predictions have a lead time of 10 to 12 h which is necessary to save life and property from damages. Accurate forecasts not only save lives but also support emergency management and mitigation. It also prevents economic losses from high impact weather. It can create major financial revenue in the energy, agriculture, transport and recreational sectors.

### Plan of work

- Weather parameters selection.
- Data collection and processing.
- Application of different methodologies (Naive Bayes, MLP, KNN, RBFN) on the processed data.
- Comparison among the results obtained from different methodologies, skill score calculation.
- Conclusion

## Data

In this study, weather data of 33 years have been considered for prediction purposes. The data of three months March–April-May (MAM) for every year from 1969 to 2002 have been chosen. These three months are known as the pre-monsoon months in India.

### Data collection

In this paper, real field meteorological data have been collected at the weather station Kolkata (22.3^0^ N/88.3^0^ E), North-East India at morning 0 GMT (6:00 am). These entire real field data are Radiosonde observational data and collected from the meteorological station (Here Kolkata, Alipore) operated by Indian Meteorological Department, Government of India (IMD). The errors were corrected at the time of observation by IMD. So, all the real field data are here error free and normalized.

North-East India generally signifies Gangatic West Bengal, Coastal region of West Bengal and Assam. The days when thunderstorms take place denoted as thunderstorm days and the days when thunderstorms did take place denoted as no thunderstorm days here in this study. The numbers of ‘thunderstorm’ days are 161 and ‘no thunderstorm’ days are 2805. In this study 100 squall days and 2600 no squall days have been considered for training purposes. These training data has been arranged in 1:26 orders and the remaining 61 squall days and 205 days has been considered as the test data set.

### Data description

In the current study different weather parameters has been considered for analysis purpose. These weather parameters are: Sun Shine Hour as X1, Pressure at freezing level (FRZ) as X2, Cloud coverage (Octa Nh) as X3 and three different dry adiabatic lapse rates at three different geo-potential heights of atmosphere as X4, X5 and X6. These parameters are essential data for the formation of thunder clouds. The main aim of this study is to predict the thunderstorms by analyzing the numerical data responsible for cloud generation. All these weather parameters are discussed in detail below.

#### Sunshine hour

The duration of the sun or the time of the sun is a climatic indicator that measures the duration of the sun in a certain phase (typically a day or a year) for a certain position on earth. It is usually articulated as an average of quite a few years. This measures the total energy delivered by sunlight over a period of time. As per the definition given by WMO in 2003 sunshine time is the period during which direct solar irradiance exceeds a threshold value of 120 watts per square meter (W/m^2^). This value is equal to the degree of solar radiation shortly after sunrise or shortly before sunset in cloudless regions. This differential heating of the atmosphere near the earth’s surface relative to the atmospheric column aloft is ultimately responsible for an instability or conditional instability. For ordinary gases such as atmospheric air that obeys the ideal gas law, parcel density at any altitude (or pressure) is determined by temperature and the buoyancy force is proportional to the temperature difference between the air parcel and its surroundings^36^. The measurement is performed by comparing the recording of the time of day using the Campbell-Stokes solar recorder with a real-time solar radiation^37^.

The sun is the ultimate source of energy for thunderstorm convection and, at a larger scale, for the general circulation of the atmosphere. Because of the clear atmospheric transparency to solar radiation, more than half of the incoming sunlight is absorbed by the Earth’s surface. A statistical data from World Meteorological Organization Standard Normal shows that mean values of Sun Shine Hour for the three months: March–April-May (MAM) of 1971–1990 are comparatively larger with the other months^38^. The most of the thunderstorm cases occur in these three months.

Solar heating drives convective currents, so thunderstorms tend to be most frequent when and where solar radiation is most intense. Hence, in most areas, thunderstorms are most frequent during the warmest hours of the day^39^. It has been observed that the phase change from water to ice or snow tends to accelerate the parcel or column upwards^40^, but synoptic acceleration is important if and only if the accumulation rate is at a frozen level or higher^41^. At first, the phase change occurs in a semi-isobaric manner and the rising air can be warmed far above the surrounding temperature. It follows that heavy rains and rainstorms can be expected to occur when such levels of accumulation are present in the pre-emitting noise when the instability phenomenon begins^40^. Therefore, sunshine hours play a vital role in the formation of thunder clouds.

#### Pressure at freezing level (FRZ)

The melting level in the troposphere where the water freezes is known as the FRZ (freezing point)^40^. It is situated at the intersection between the 0 °C isotherm and the temperature ratchet. An FRZ level with a pressure level of 650 mb or closer to the surface, in severe weather conditions, will usually carry large hailstones. This will have more time to grow in cold air and will have less time to melt as it falls to the surface^42^.As a result of the convection process the hot air rises, transferring temperature to the upper levels of the atmosphere from the Earth's surface. The water vapor they contain begins to cool, release heat, condense and form clouds^40^. The pressure at freezing level is measured using aneroid barometer. It is a device for measuring atmospheric pressure without the use of fluids^43^.

#### Cloud coverage (Octa Nh)

The measure of atmospheric moisture is indicated by the cloud content of the upper atmosphere. The most important ingredient to form the thunder cloud is this atmospheric moisture. The amount of moisture in the upper air increases with the increase in cloud content. The Cloud coverage is measured by Ceilometers^44^.

#### Dry adiabatic lapse rates

The dry bulb temperature difference between two consecutive levels at different geo-potential heights of the atmosphere is the measure of the dry adiabatic lapse rate (dT/dZ). In this study four heights (dZ) of the atmosphere have been considered. These heights are (a) 700 hpa and 600 hpa (approximately 3100 to 4500 m), denoted by X4 (b) 600 hpa and 400 hpa (approximately 4500 to 7500 m), denoted by X5, and (c) 400 hpa and 300 hpa (approximately 7500 to 9600 m), denoted by X6. The temperature differences (dT) between these consecutive heights have been taken into account. The change in temperature is measured by Thermistors. Thermistors are temperature-dependent resistors, changing resistance with changes in temperature. They are very sensitive and react to very small changes in temperature^45^.

The dry adiabatic lapse rate of the atmosphere is the measure of the conditional instability^39^. The conditional instability in the atmosphere is the reason for presence of moisture which would carry out to the upper atmosphere from the surface level to form thunder clouds^36^. A statistics from world Meteorological Standard Organization reflects that mean value of the dry bulb temperature remains the maximum during the March–April-May. The thunderstorms occur in these three months. It can be observed from the statistical data that the mean values of dry bulb temperature during other months (except March–April-May) were comparatively lower^46^.

## Methodologies

### Naïve Bayes classifier

Naïve Bayes classifier is a supervised learning algorithm and is utilized for the solution of classification problems^47^. It is based on the Bayes theorem^47^. The Bayes decision theorem is a fundamental statistical approach to recognize a pattern^47^. It is preferable for high dimensional training data sets and quick prediction. Bayes theorem states that,

1 $$P\left( {AB} \right) = \frac{{P\left( {BA} \right)P\left( A \right)}}{P\left( B \right)}$$

where,

P (A|B) is posterior probability: Probability of hypothesis A on the observed event B.

P (B|A) is likelihood probability: Probability of the evidence given that the probability of a hypothesis is true.

P (A) is prior probability: Probability of hypothesis before observing the evidence.

P (B) is marginal probability: Probability of Evidence.

The expression P (A) refers to the probability that event A will occur. P (A|B) stands for the probability that event A will happen; given that event B has already happened. In other words, P(A|B) is the probability of the object belonging to class A i.e., the probability of the attribute values (predictors which are Sun Shine Hour, Pressure at freezing level, Cloud coverage and three different dry adiabatic lapse rates at three different geo-potential heights of atmosphere) B belonging to class A (squall or no squall days)^48^.

Here is the algorithm for Naive Bayes procedure:

- Convert the training dataset into corresponding frequency tables.
- Generate likelihood table by finding the probabilities of the mentioned parameters.
- Then the Bayes theorem is used to compute the posterior probability.

Naive Bayes is straightforward probabilistic classifier^49^. This often gives reasonable solution in many real-world problems^50^. Despite of its unrealistic independence hypothesis, the Naïve Bayes classifier is astonishingly successful in exercise^50^. The performance of Naïve Bayes classification is fairly good, as evidenced by the many experimental studies^51^. In this study Table 1 from the result Sect. 4 shows that Naïve Bayes classification yields 85.25% correct prediction for ‘squall days’ and 86.34% correct prediction for ‘no squall days’.

**Table 1:** Correct prediction of ‘squall days’ and ‘no squall’ days using Naïve Bayes classification.

Training data for squall days = 100	Training data for no squall days = 2600	
	Squall (total no. of test data = 61)	No-squall (total no. of test data = 205)
Six variables	52, 85.25%	177, 86.34%

### K-nearest neighbor (K-NN)

K Nearest Neighbor (K-NN) is one of the familiar names in the field of data classification^52^. The K-NN algorithm was successfully applied by Cover in1967. This is a straightforward algorithm that reserves all existing cases and classifies new cases created based on the amount of vicinity. The K-NN determines the way that which of the points from the training sets is similar enough to be considered^53^.The k value in the k-NN algorithm defines how many neighbors will be checked to determine the classification of a specific query point. For example, if k = 1, the instance will be assigned to the same class as its single nearest neighbor^54^.The principle of the algorithm is established on a comparison between a given testing data point and training data points^52^. This sorts out the training data points which are in close vicinity (neighbors) with test data points, and then predicts the corresponding class label of these neighbors^53^. It can be said that neighbors are measured by a distance or dissimilarity measure that can be computed between samples based on the independent variables^52^. KNN is a non-parametric procedure to classify items built on closest training instances in the feature space^53^. One of the best examples of instance-based learning or lazy learning is KNN^52^. Here, the function is estimated locally and all calculations are delayed until classification^8^. In the classification stage, K is a user-defined constant, and these are not previously labeled^53^. Here in this study K have been chosen as 1, 3, and 5. All training data vectors have a class label^53^. The training stage of the algorithm contains only loading the feature vectors and class labels of the training objects^55^. The similarity measure has been considered between each data vector of test data set with each data vector of training data set. Similarity between two vectors can be defined as,*p* = *(p1, p2,…, …., pγ), q* = *(q1, q2,… , qγ)* is defined as,

2 $$\frac{{\mathop \sum \nolimits_{i = 1}^{\gamma } p_{i} q_{i} }}{{\sqrt {\left( {\mathop \sum \nolimits_{i = 1}^{\gamma } p_{i}^{2} \mathop \sum \nolimits_{i = 1}^{\gamma } q_{i}^{2} } \right)} }}$$

**Figure 1:**
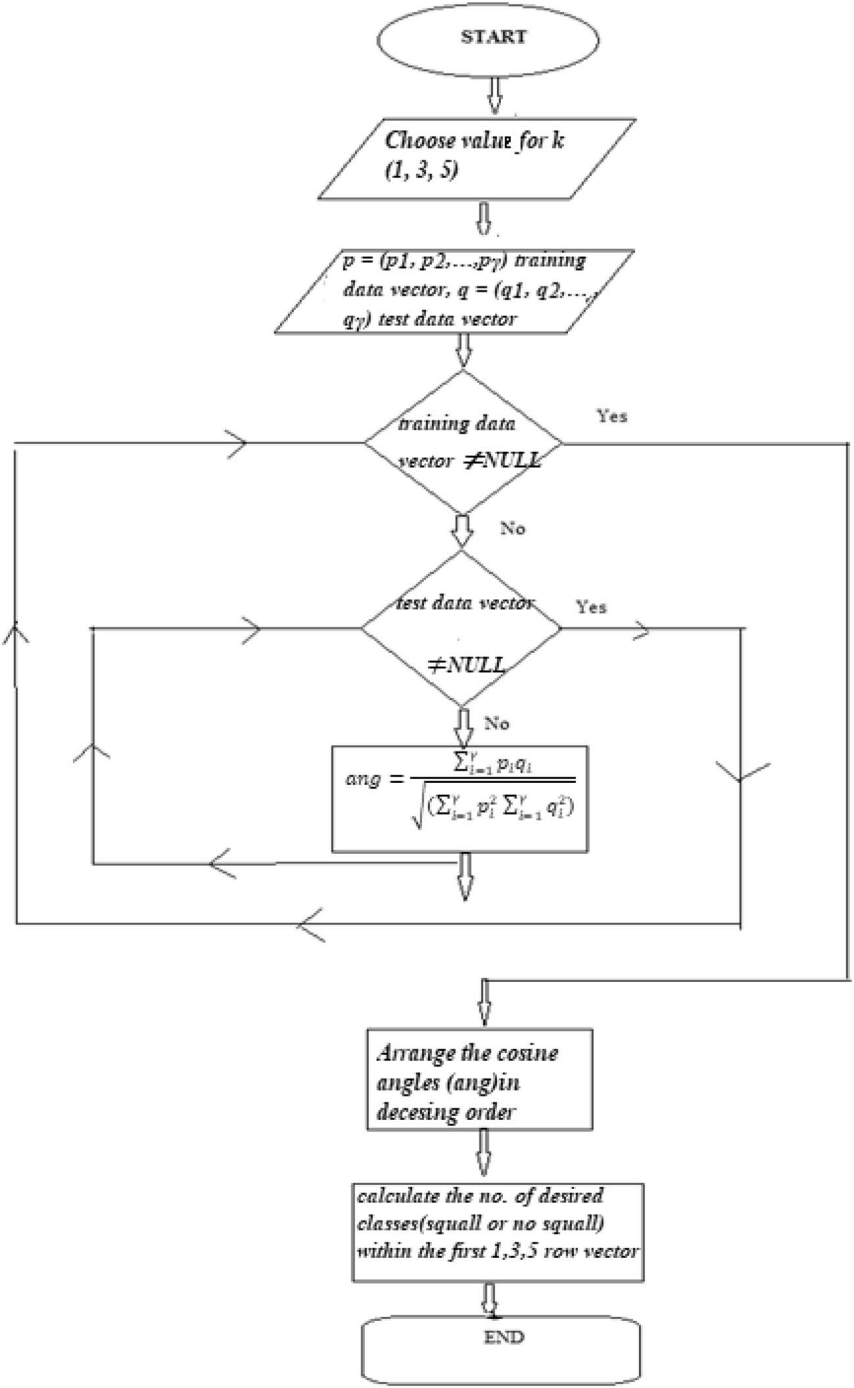
Flowchart for KNN methodology.

Here p corresponds to training data vector and q corresponds to test data vector. Here value of *γ* is 6 since numbers of parameters are six. Here p1 and q1 corresponds to Sun Shine Hour (variable X1), p2 and q2 corresponds to Pressure at freezing level (FRZ, variable X2), p3 and q3 corresponds to Cloud coverage (Octa Nh, variable X3), p4 and q4 (variable X4), p5 and q5 (variable X5), p6 and q6 (variable X6) corresponds to three different dry adiabatic lapse rates at three different geo-potential heights of atmosphere respectively. The flowchart for KNN has been depicted in the Fig. 1.

The cosine angle between two vectors indicates the similarity measure between them^52^, which will be greater if the angle value is smaller. The similarity measure indicates the vicinity of each data vector of the test set with each data vector of the training set. These cosine angles are arranged in decreasing order. The result of Table 2 from section "Result" shows that 3NN gives the most promising result in comparison with 1NN and 5NN. The 88.52% correct prediction for ‘squall days’ and 87.8% correct prediction for ‘no squall days’ were obtained by applying 3NN.

**Table 2:** Correct prediction of ‘squall days’ and ‘no squall’ days using K Nearest Neighbor Method.

K = 1		K = 3		K = 5	
Training data for squall days = 100	Training data for no squall days = 2600	Training data for squall days = 100	Training data for no squall days = 2600	Training data for squall days = 100	Training data for no squall days = 2600
Squall (total no. of test data = 61)	No-squall (total no. of test data = 205)	Squall (total no. of test data = 61)	No-squall (total no. of test data = 205)	Squall (total no. of test data = 61)	No-squall (total no. of test data = 205)
52, 85.25%	174, 84.88%	54, 88.52%	180, 87.8%	50, 81.97%	175, 85.37%

### Multilayer perceptron

One of the most widely used empirical approaches for weather prediction is artificial neural network^56^. A three-layered Multilayer Perceptron (MLP) network has been applied to the above mentioned six weather variables. It consists of an input layer, one hidden layer and an output layer. As such, neural networks are extremely complex^51^. The ANN (Artificial Neural Network) reduces the error using a variety of algorithms. This produces an approximated value that is close to the real value^57^. One of the most promising branches of artificial intelligence is neural network. It has many applications in the field of space weather prediction such as forecasting geomagnetic storms^58^ and solar flairs^59^. A single layer perceptron with one input produces decision regions under the form of semi planes^51^. The addition of one layer causes every neuron to act as a standard perceptron for the outputs of the neurons in the anterior layer. Therefore, the output of the network can evaluate convex decision regions, which results from the intersection of the semi planes produced by the neurons^60^. Sequentially, a three-layer perceptron can create arbitrary decision areas^60^.

#### Learning phase

In the learning phase of the Multilayer Perceptron, the ‘occurrence’ of storm days is represented by a value of 1 and ‘no occurrence’ of the storm days is represented by the value of 0. Every unit of every layer is associated with every unit of the next layer by the connection weights^4^. The sigmoid function is chosen as the transfer function which acts as a nonlinear activation function. Two different modes of learning the weights of an MLP exist. These are Batch mode learning and On-line learning. Here, On-line method of learning the weights is considered^51^.

#### Feed forward stage

The multilayer perceptron is the neural network model that is commonly known and most frequently used in different types of applications. Generally, the signals are transferred within the network unidirectionally from input to output. The initial part of this architecture is called the feed forward stage of the network^60^. In this stage each node (say *i*) in layer *α* is joined to each node (say *j*) in the next layer (*α* + *1*), with a connection weight represented by $W_{ij}^{(\alpha )}$^60^. Let *S*_i_ be the i-th input node in the input layer. Then the activation unit for the hidden layer is *Y*_*i*_, which is the output from the nodes of the input layer. *Y*_*i*_is the total input received for the j-th node in the hidden layer.

3 $$Yi = \mathop \sum \limits_{i = 1}^{n} S_{i} W_{ij}$$

The output from the j-th node of the hidden layer is *Y*_*j*_. A transfer function is used to obtain this^51^.

4 $$Y_{j} = \frac{1}{{1 + \exp \left( { - Y_{i} } \right)}}$$

This is valid for every layer.

#### Connection weights

Connection weights (*W*’s) are adjusted to trivial random values in the range (− 0.5 to 0.5)^4^. A threshold value is correspondingly presumed. The weight values are altered in back propagation stage of the learning of the model until the error is reduced^4^. The test data is validated by these modified weights. The gradient descent technique is mainly used in back propagation process to modify the weights. It is used to minimize the chances of becoming trapped in local optimal points or saddle points of the network^51^.

#### Error

The error function is measured by the mean square error. This is given as follows,

5 $$E = \frac{{\mathop \sum \nolimits_{i = 1}^{2} \left( {o_{j} - e_{j} } \right)^{2} }}{2}$$

The expected output (*e*_*j*_) for each data point in the training set is recognized^51^. For a specific scenario the real output value for the j-th node in the output layer is *o*_*j*_^51^. The error has to be reduced during the training time using back propagation. Iteration is continued until the error is reduced approximately 0.005 to 0.001^4^.

#### Back propagation of error

In the present case, the back propagation rule is applied to the set of training patterns of data. This rule basically uses the gradient descent technique for changing the weights. The main aim is to arbitrate the modification of weight representation of an input–output pattern pair. Since given data can be used numerous times during training, let us use the index *m* to denote the presentation step for the training pair at step m^51^. For training a multilayer feed-forward neural network, the subsequent approximation is used by applying the gradient descent along the error surface^51^ to determine the increase in the weight connecting units *j* and *i*:

6 $$\Delta w_{ij} \left( m \right) = - \eta \frac{\delta E\left( m \right)}{{\delta W_{ij} }}$$

where *η* = 0.01 is the learning rate parameter.

*E(m)* denotes the measure of performance, the negative derivative of *E(m)* with respect to the weight *Wij* can be defined as the negative gradient of *E(m).*

#### Updation of weights

The weight update is given by,

7 $$W_{ij} \left( {m + 1} \right) = W_{ij} \left( m \right) + \Delta W_{ij} \left( m \right)$$

**Figure 2:**
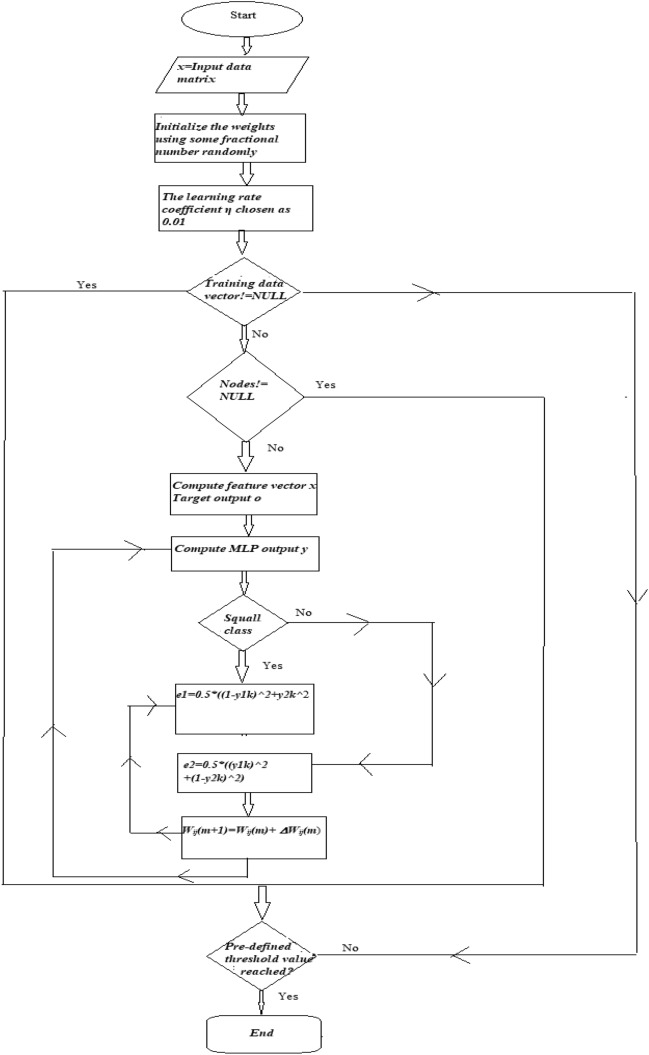
Flowchart for MLP methodology.

The modified weights are used in the test dataset to validate the outputs^51^. Sometimes, if the number of iterations becomes too much large or if the classifications on the test set are insufficient, the error may not be minimized^51^. In such cases, the architecture of MLP is to be modified by modifying the number of nodes in the hidden layer or by changing the number of hidden layers^4^. MLP include too many parameters because it is fully connected. Each node is connected to another in a very dense web — resulting in redundancy and inefficiency^61^.Here in this study three layered MLP has been considered. These are 6–3-2, 6–4-2, and 6–5-2. Here the first layer represents input layer, second layer represents hidden layer and third layer represents output layer. Table 3 from section "Result" shows that applying MLP gives 91.8% correct prediction for ‘squall days’ and 89.27% correct prediction for ‘no squall days’ obtained. The flowchart for MLP has been depicted in the Fig. 2.

**Table 3:** Correct prediction of ‘squall days’ and ‘no squall’ days using MLP.

Design of the Network	No. of correctly classified and % of accurate points for ‘squall storm’ days in test data sheet	No. of correctly classified and % of accurate points for ‘no squall storm’ days in test data sheet	No. of correctly classified and % of accurate points for in data sheet. Data set for both ‘squall storm ’days and ‘no squall storm ’ days are considered
	Total no. of squall storm days = 61	Total no. of no squall days = 205	Total no. of days = 266
6–3-2	53, 86.88%	177, 86.34%	230, 86.47%
6–4-2	56, 91.8%	183, 89.27%	239, 89.85%
6–5-2	50, 90.16%	183, 89.27%	233, 87.59%

### Radial basis function network

Artificial Neural Network (ANNs) offers a methodology for explaining different kinds of nonlinear problems that are complex to solve by conventional methodologies^62^. There are several types of ANN (Artificial Neural Network) and the Radial Basis function is one of them. Radial Basis Functional Networks (RBFNs) are non-linear layered feed forward networks^63^. It can implement arbitrary non-linear transformations of the input space. There are different applications of RBFNs^64^. The RBFNs are most effective for prediction purposes such as weather prediction, modeling, pattern recognition, and image compression^64,65^. It contains three different layers: input layer, hidden layer and output layer. The hidden layer is multidimensional and defined as radial counters^47^.

Each hidden unit is defined as a radial center and every center represents one or some of the input patterns^66^. The network is known as a ‘localized receptive field network’^64^. The hidden units in RBFN have Gaussian activation functions as follows:

8 $$\emptyset_{i} \left( X \right) = \varphi \left( {\left| {\left| {x t_{i} } \right|} \right|} \right)$$

where $\left|\left|{\varvec{x}}\boldsymbol{ }\boldsymbol{ }{{\varvec{t}}}_{{\varvec{i}}}\right|\right|$ denotes the Euclidean norm function and *φ* is the RBF neuron activation function. The input vector is denoted by *x* i.e., the input weather data and *t*_*i*_ denote the neuron’s prototype vector. The approximation of output, by an RBF will be denoted by ŷ_t_.

9 $$\mathop Y\limits^{ \wedge }_{t} = \mathop \sum \limits_{i = 1}^{m} \lambda_{i} \emptyset \left( {x_{i} ,\;C_{i} ,\;\sigma_{i} } \right)$$

This approximation will be the weighted sum of m Gaussian kernels $\boldsymbol{\varnothing }$**:**

10 $$\emptyset \left( {x_{i} ,\;C_{i} ,\;\sigma_{i} } \right) = \exp \left( {\frac{{ - x - x_{t} }}{{\sqrt 2 \sigma_{t} }}} \right)^{2}$$

Gaussian kernels are used to determine the complexity of RBFN. The various parameters to specify are the positions of the Gaussian kernels (Ci)^66^. The second parameter to be chosen is the standard deviation (or width) of the different Gaussian kernels σi. The last parameter is denoted by the multiplicative factor λi^66^.

The hidden layer in RBF is of high dimension, which has a different purpose than in a multilayer feed forward network^66^.The radial distance di, between the input vector x and the center of basis function Ci is computed for each unit i in the hidden layer as follows:

11 $$d_{i} = x - C_{i}$$

12 $$y = f\left( x \right) = \mathop \sum \limits_{i = 1}^{k} w_{i} \varphi_{i} \left( {x - C_{i} } \right)$$

**Figure 3:**
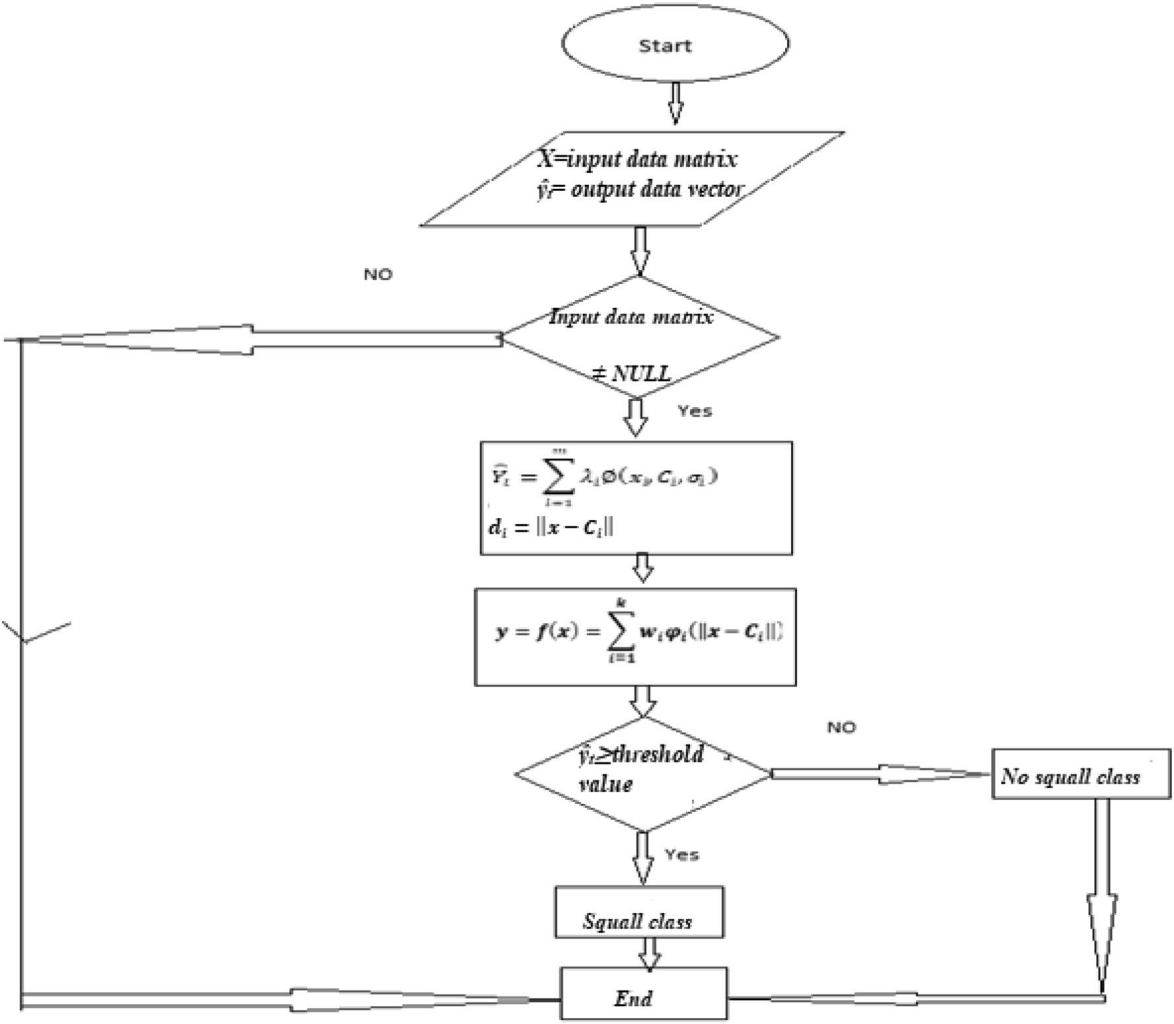
Flowchart for RBFN methodology.

Here, f denotes nonlinear activation function, x denotes input, φ_1_, φ_2_, …, …, φ_m_ denotes RBF centers in the input vector space^63^; every neuron in the hidden layer has its adjoining center, X denotes the input vector, k denotes the total number of hidden layer neurons and i denotes the j-th node in the hidden layer^63^. Although the training is faster in RBF network but classification is slow in comparison to Multi layer Perceptron due to fact that every node in hidden layer have to compute the RBF function for the input sample vector during classification^67^.Here in this study three layered RBFN has been considered. These are 6-7-1, 6-8-1, and 6-9-1. Here the first layer represents input layer, second layer represents hidden layer and third layer represents output layer. The flowchart for RBFN has been depicted in the Fig. 3.

Table 4 from section "Result" shows that RBFN gives 95.08% correct prediction for squall days and 94.15% correct prediction for no squall days.

**Table 4:** Correct prediction of ‘squall days’ and ‘no squall’ days using six weather variables using RBFN.

Design of the Network	No. of correctly classified and % of accurate points for ‘squall storm’ days in test data sheet	No. of correctly classified and % of accurate points for ‘no squall storm’ days in test data sheet	No. of correctly classified and % of accurate points for in data sheet. Data set for both ‘squall storm ’days and ‘no squall storm ’ days are considered
	Total no. of squall storm days = 61	Total no. of no squall days = 205	Total no. of days = 266
6–7-1	56, 91.8%	190, 92.68%	246, 92.48%
6–8-1	58, 95.08%	193, 94.15%	251, 94.36%
6–9-1	58, 95.08%	190, 92.68%	248, 93.23%

## Result

Here results of four different methodologies have been represented. A total of 61 squall and 205 no squall days were chosen as test data randomly from 1969 to 2002 from the three months of March–April-May (MAM). There was a strong squall line over the sky of Kolkata (22.3°N/88.3°E) on these 61 squall days and severe thunderstorm occurred. There was no thunderstorm activity observed during these205 no squall days over Kolkata (22.3°N/88.3°E). Here in this study WEKA 3.8.5 has been used as a common package tool to perform Naïve Bayes, K-NN, MLP and RBFN. This is free software and the operating platform is Windows 7.

The result of Table 1 shows that the application of Naïve Bayes methodology on the above-mentioned sample days produces 85.25% correct prediction for ‘squall days’ and 86.34% correct prediction for ‘no squall days’.

Table 2 shows that KNN yields better results on these six weather variables in comparison with Naïve Bayes methodology. The result of Table 2 shows that 3NN gives the most promising result in comparison with 1NN and 5NN. The 88.52% correct prediction for ‘squall days’ and 87.8% correct prediction for ‘no squall days’ were obtained by applying 3NN. Table 2 shows that KNN yields better results on these six weather variables in comparison with Naïve Bayes methodology.

Table 3 shows that applying MLP 91.8% correct prediction for ‘squall days’ and 89.27% correct prediction for ‘no squall days’ has been obtained.

The most promising results yield from the application of RBFN (Table 4) on these six weather variables. RBFN gives 95.08% correct prediction for squall days and 94.15% correct prediction for no squall days.

It can be concluded from Table 5 that among these four methodologies RBFN gives the lowest misclassification rate for squall days.

**Table 5:** Misclassification rate comparison among four methodologies applied to six weather variables.

Naïve Bayes		MLP		KNN		RBFN	
Squall class	No squall class	Squall class	No squall class	Squall class	No squall class	Squall class	No squall class
0.15	0.14	0.08	0.11	0.11	0.12	0.05	0.06

The Heidke Skill Score (HSS) has also been applied here for the purpose of forecast which is a measure of skill. The Heidke Skill Score (HSS) is a skill score for categorical forecasts^68^. It is defined as follows,

$${\text{HSS = }}\frac{{\left( {\left( {{\text{Hit + Correct}}\;{\text{ negatives}}} \right){ - }\left( {{\text{Expected}}\;{\text{ correct}}} \right)_{{{\text{random}}}} } \right)}}{{{\text{N - }}\left( {{\text{Expected }}\;{\text{correct}}} \right)_{{{\text{random}}}} }}$$

where, $${\left(\text{expected correct}\right)}_{\text{random}}\text{=}\frac{1}{{\text{N}}}\left[\left(\text{hit+misses}\right)\left(\text{hit+false alarms}\right)\text{+}\left(\text{correct negatives+misses}\right)\left(\text{correct negatives+false alarms}\right)\right]$$

Here, N denotes total number of test data; the term hit represents event forecast to occur, and did occur; miss denotes event forecast not to occur, but did occur; false alarm represents event forecast to occur, but did not occur and correct negative represents event forecast not to occur, and did not occur.

It can be analyzed from Table 6 (contingency table) what types of errors are being made.

**Table 6:** Contingency Table.

Forecast	Observed		
	Yes	No	Total
Yes	Hits	False alarms	Forecast yes
No	Misses	Correct negatives	forecast no
Total	Observed yes	Observed no	Total

Here ‘yes’ indicates squall days and ‘no’ indicates no squall days.

The HSS for the different methodologies has been obtained from the following contingency tables.

Therefore it can be obtained from the Table 7 that the Heidke Skill Score (HSS) for Naïve Bayes is 0.66.

**Table 7:** The contingency table for Naïve Bayes.

Forecast	Observed		
	Yes	no	Total
Yes	52	28	80
No	9	177	186
Total	61	205	266

Therefore it can be obtained from the Table 8 that the Heidke Skill Score (HSS) is 0.62 for 1 NN, 0.69 for 3NN and 0.61 for 5NN respectively.

**Table 8:** The contingency table for 1NN, 3NN and 5NN.

Forecast	1NN			3NN				5NN			
	Observed			Observed				Observed			
	Yes	No	Total	Forecast	yes	no	Total	Forecast	Yes	No	Total
Yes	52	31	83	Yes	54	25	79	Yes	50	30	80
No	9	174	183	No	7	180	187	No	11	175	186
Total	61	205	266	Total	61	205	266	Total	61	205	266

Therefore it can be obtained from the Table 9 that the Heidke Skill Score (HSS) for MLP is 0.74.

**Table 9:** The contingency table for MLP.

Forecast	Observed		
	Yes	No	Total
Yes	56	22	78
No	5	183	188
Total	61	205	266

Therefore it can be obtained from the Table 10 that the Heidke Skill Score (HSS) for RBFN is 0.85. The HSS measures the fractional improvement of the forecast over the standard forecast. HSS 0 means no skill, and a perfect forecast obtains a HSS of 1. Here RBFN exhibits the HSS value as 0.85 which is close to 1. Therefore it can be said that RBFN gives the best result among the other three methodologies here.

**Table 10:** The contingency table for RBFN.

Forecast	Observed		
	Yes	No	Total
Yes	58	12	70
No	3	193	196
Total	61	205	266

## Conclusion

The study here predicts severe thunderstorms using both statistical and ANN methodologies on numerical weather data. The numerical simulation depends on the volume of the input data set^69^. Neural network classifiers have been attractive alternatives to conventional classifiers by numerous researchers^7^. The methodologies that are considered here have advantages and disadvantage both. The ANN methodologies produce output even with incomplete information. The ANN methodologies have much more fault tolerant capability^70^. The MLP and RBFN methodology both work well for large amount of data. In case of MLP there is loss of non convex function when there is more than one local minimum^70^. Although the training is faster in RBF network but classification is slow in comparison to Multi layer Perceptron due to fact that every node in hidden layer have to compute the RBF function for the input sample vector during classification^71^. RBF network works more effectively on noised input data set^71^. The KNN on the other hand gives better classification on rare events; it performs well for multi-classification issues^72^. The KNN shows poor result if the sample size is not properly balanced^72^. The choice of the value of K is one of the most crucial factors for correct prediction. The Naive Bayes methodology is easy to implement and the training is fast. The main disadvantage of Naive Bayes methodology is conditional independence assumption which does not always hold. In most situations, the feature show some form of dependency^47^. Different previous studies has showed that the application of MLP, KNN on weather parameters like moisture difference and wind shear can produce very effective result for thunderstorm prediction purpose^52^,^52^. Therefore, here in this study some different kind of weather parameters has been considered for thunderstorm prediction purpose. There are many studies that used both statistical and ANN methodologies to predict severe thunderstorm. But there is no notable study where RBFN and Naive Bayes methodologies have been used for severe thunderstorm prediction successfully. RBFN gives more accuracy and builds the model faster than MLP. The aim of this study is not only to predict severe thunderstorm correctly but also to establish an effective comparative findings among ANN and statistical methodology. The present study can be extended in future by the analysis of cloud imageries for thunderstorm prediction purpose. Table 5 shows that among the four methodologies, RBFN exhibits the minimum misclassification rate. In this work the best result have been obtained by applying RBFN (ANN methodology) among the other methodology that have been used on the weather data. It can be concluded that the Naïve Bayes methodology yields less promising results for ‘squall’ days in comparison with the other three methodologies. Overall both the statistical and ANN methodologies give more than 80% correct prediction for severe thunderstorm in this study. Generally, thunderstorms occur in the North-East India during the evening. Lead time is the period between the time of prediction and occurrence of the event. Thunderstorm is a catastrophic event, generating in the early morning and occurring in the evening time. So, accurate prediction with enough lead time is very pertinent to protect the social life. Sufficient lead time is also helpful for local Government to make the people alert and to take safety measures for the people. Therefore, in this study 10–12 h as the lead time has been considered.

## Data Availability

The datasets used and/or analysed during the current study are available from the corresponding author on reasonable request.
